# A Pilot Study to Explore a Correlation between Inflammatory Markers and the Wound Healing Rate in Diabetic Patients

**DOI:** 10.3390/medicina58030390

**Published:** 2022-03-06

**Authors:** Yukwan Song, Yongkyu Jo, Jeongeun Sohn, Robert Kim

**Affiliations:** 1 Department of Plastic and Reconstructive Surgery, Soonsoo Hospital, Hwaseong-si 18617, Gyeonggi, Korea; 2 Department of Anesthesiology and Pain Medicine, Cheju Halla General Hospital, Jeju 63127, Korea; pudding7459@naver.com; 3 Department of Medical and Pharmaceutical Affairs, Doctor CONSULT, Seoul 06296, Korea; mcgcompany@naver.com

**Keywords:** wounds and injuries, wound healing, diabetes mellitus, inflammation

## Abstract

*Background and objectives*: We examined whether there is a significant correlation between inflammatory markers and the wound healing rate (WHR) in diabetic patients. *Materials and Methods*: A total of 60 patients were divided into two groups depending on the completion of wound healing (WH) at 5 weeks: the early WH group (period of WH < 5 weeks; *n* = 27) and the late WH group (period of WH > 5 weeks; *n* = 33). The baseline characteristics and wound measurements were compared between the two groups. To identify the correlation between inflammatory markers (e.g., white blood cell counts (WBCs), serum C-reactive protein (CRP) levels and erythrocyte sedimentation rate (ESR)) and WHR, we performed a Pearson correlation analysis. *Results*: The WHR was 8.06 ± 4.02 mm^2^/day in the early WH group and 2.71 ± 0.88 mm^2^/day in the late group. This difference reached statistical significance (*p* < 0.001). Moreover, WBC counts were significantly higher and serum levels of CRP and ESR were significantly lower in the early WH group than in the late group (*p* = 0.027, 0.036 and 0.043, respectively). *Conclusions*: Our results indicate that WBC as well as serum CRP and ESR levels have a significant correlation with WHR in diabetic patients.

## 1. Introduction

Skin is the largest human organ; it is composed of epidermis and dermis, and serves as the first line of protection against external invasion [1,2]. Once damaged, wounds can be categorized as normal or chronic [3]. A wound is a loss of skin integrity that arises from injury or disease. It should be healed by repair and regenerative mechanisms because intact skin is an essential element for protecting organisms against the environment, for which restoration of the pre-injured form and skin functions serves as the primary goal of wound healing [4].

Wound healing (WH) is a dynamic biological process, whose onset is noted immediately after tissue injury. The normal WH process involves four overlapping but independent phases [3,5]. First, immediately after the onset of injury, growth factors (GFs) and cytokines are released during the hemostasis phase [6]. Second, hemostasis is followed by inflammation within hours; immune cells such as neutrophils are infiltrated into the site of injury to scavenge bacteria and damaged matrix proteins [7]. Third, inflammation is followed by proliferation. During proliferation, diverse types of GFs, such as platelet-derived growth factor (PDGF), vascular endothelial growth factor (VEGF) and fibroblast growth factors (FGFs), are released and then used to promote epidermal repair and angiogenesis. This leads to the synthesis of granulation tissue [8]. Fourth, proliferation is followed by remodeling of the extracellular matrix (ECM), during which type III collagen is replaced with type I collagen in the dermis. This results in the formation of scar tissue that is abundant in collagen fibers [9].

Any derangements in the phases of the WH process may cause prolonged or delayed WH. Causes of prolonged or delayed WH include local factors (e.g., bacterial infection and lower oxygen tension) and systemic factors (e.g., damage to the nervous system, metabolic abnormalities and aging) [10]. Chronic wounds may last for months or even years when there are no treatment effects of conservative wound care, thus disturbing rehabilitation therapy. This may impose a social, medical and economic burden on patients [11]. Moreover, a difficulty in predicting the time to healing poses a therapeutic dilemma, even to expert wound care practitioners, who may feel it necessary to change treatment modalities for patients [12].

To date, laboratory investigations and clinical studies have yielded information about critical factors that are involved in both normal and impaired WH. Physiological responses to tissue injury in healthy individuals are characterized by timely healing with full re-epithelialization, resolution of drainage and restoration of functions [13]. However, this intricate sequence is not seen in patients with chronic wounds; such physiologic events stall in some phases of WH without progression to the following phase. A lack of progression may arise from a lack of certain biological substances needed for the affected area as well as an inability to recruit certain types of cells [14].

It is known that patients with diabetes mellitus (DM) are vulnerable to chronic low-grade inflammatory responses; elevated levels of inflammatory markers such as C-reactive protein (CRP), white blood cell (WBC) counts, interleukin (IL)-1β, IL-1 receptor antagonist (IL-1RA), IL-6, IL-8, IL-18, monocyte chemoattractant protein-1 (MCP-1), interferon-γ-inducible protein-10 (IP-10), haptoglobin and fibrinogen are seen prior to the onset of type 2 DM (T2DM) [15]. Diabetic wounds (DWs), defined as chronic wounds or lesions that take a long time to heal, fail to heal or recur (e.g., leg ulcer or DFU) in patients with DM, remain a major concern in that DM plays a role in delaying the WH process. This has long-term detrimental effects on the quality of life, morbidity and mortality of patients with DM. Patients with DWs commonly present with delayed acute wounds and chronic wounds, which is characterized by a persistent presence of inflammatory responses that are closely associated with an impaired formation of mature granulation tissue and a decrease in the tensile strength of the wound. Presumably, this might arise from vascular damages resulting in ischemia [16,17,18,19].

To date, several measures of the wound healing rate (WHR) have been introduced for the purposes of quantifying the effects of diverse treatment options in patients with wounds. Due to differences in definitions of WHR, however, it has become difficult to compare outcomes between treatment modalities [20]. Moreover, Goto et al. reported that WHR has a significant correlation with diverse factors, including CRP [21].

Given the above background, we conducted this retrospective study to examine whether there was a significant correlation between inflammatory markers and WHR in a cohort of patients with T2DM.

## 2. Materials and Methods

### 2.1. Study Design and Setting

In the current study, we analyzed a total of 60 patients (*n* = 60) who had been treated at our medical institution during an 18 month period ranging from January 2019 to August 2020. We included men and women aged 18 years or older, those with a confirmed diagnosis of T2DM, those with full-thickness or nearly full-thickness skin defects and those who underwent simple dressing. We excluded patients with wounds with eschar, current smokers and those with low serum albumin levels, all factors that may affect the WH process [22,23,24].

We therefore evaluated a total of 60 patients in the current study; it was approved by the Korea National Institute of Bioethics Policy. Informed consent was waived due to the study’s retrospective nature.

### 2.2. Treatment Protocol and Laboratory Examination

On admission, the patients with systemic signs and symptoms suggestive of infected wounds, such as redness, heat, induration, swelling and, if any, purulent discharge, received clinical and laboratory examinations. Moreover, patients were also evaluated for the measurement of WBC counts, CRP levels, erythrocyte sedimentation rate (ESR) as well as albumin, protein, glycated hemoglobin (HbA1c) and blood glucose (BG) levels [25,26].

For laboratory examination, blood samples were obtained from each patient and WBC counts were counted accordingly. In addition, serum CRP levels and ESR were also measured. Serum CRP levels were measured by a highly sensitive sandwich enzyme-linked immunosorbent assay (ELISA) technique using anti-human-CRP goat antibody (primary), rabbit one (secondary) and horseradish peroxidase-conjugated anti-rabbit-IgG goat IgG (tertiary) [27]. Furthermore, ESR values were determined using the standard Westergren method [28].

### 2.3. Measurement of Wound Characteristics including WHR

In the current study, wound measurements were the wound’s surface area, the length of its edge and the WHR, and were obtained as previously described [29,30].

**Figure 1:**
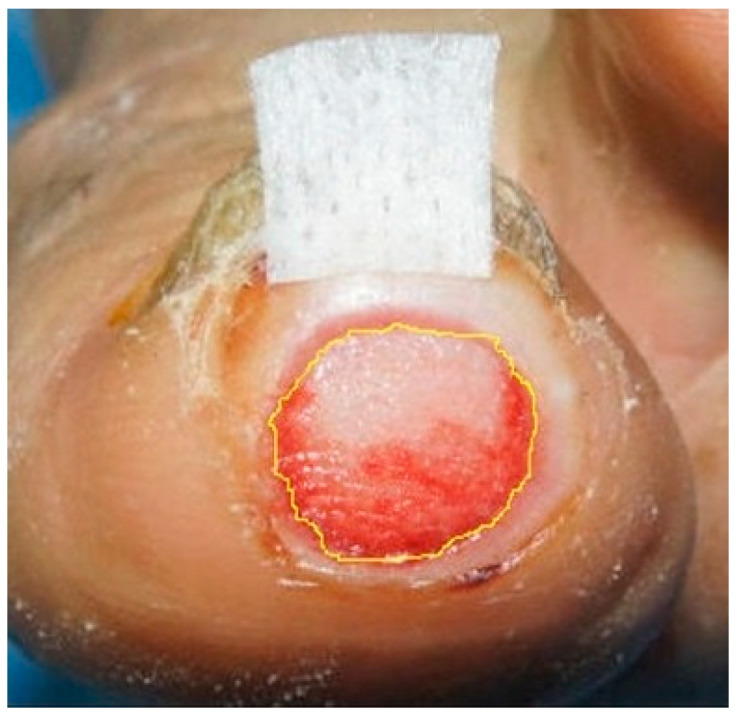
Measurement of the wound size. To measure the size of the wounds, we placed an adhesive marker next to them.

After placing an adhesive marker next to the wounds, we took pictures of them and then analyzed the photographs using Image J™. After marking the edge of wounds, we calculated not only the number of pixels falling under the square adhesive marker but also the number of pixels taken up by the marked wounds. Based on the dimensions of the square (16 mm^2^), we derived the actual size of the area of the marked wounds (Figure 1) [29,30].

To calculate the WHR, we initially set a reference for comparison of the surface area of wounds at 100 mm^2^ and divided it by its number of pixels. The result serves as the conversion factor (A). That is, 100 mm^2^/(the number of pixels of the reference for comparison of the area of wounds) served as A. Then, we calculated the actual area of wounds as (the number of pixels of the area of wounds) × A. Finally, we defined the WHR (mm^2^/day) as a daily decrease in the actual surface area of the wound. Then, we monitored changes in the WHR during a 7 week period [31,32].

### 2.4. Patient Evaluation and Criteria

For the current study, we performed a retrospective review of medical records and thereby evaluated baseline characteristics of the patients. These included age, sex, body mass index (BMI), the shape of wounds, the location of wounds (upper extremities, trunk and lower extremities) and the initial size of wounds. We also evaluated laboratory findings of the patient data.

The patients were divided into two groups: early WH and late WH. Early WH was defined as a decrease in the total area of wounds by more than 50% within the first 2 weeks, and wound closure within 5 weeks; reported values refer to the percentage of gap closure within a fixed 5 week period [33,34]. Then, baseline characteristics, wound measurements and laboratory findings of the patients were compared between the two groups.

### 2.5. Statistical Analysis

All data are expressed as mean ± SD (standard deviation). To identify the correlation between inflammatory markers and WHR, we performed a Pearson correlation analysis. Statistical analysis was conducted using SPSS 17.0 for Windows (SPSS Inc., Chicago, IL, USA). A *p*-value of <0.05 was considered statistically significant.

## 3. Results

### 3.1. Baseline Characteristics of the Patients

**Figure 2:**
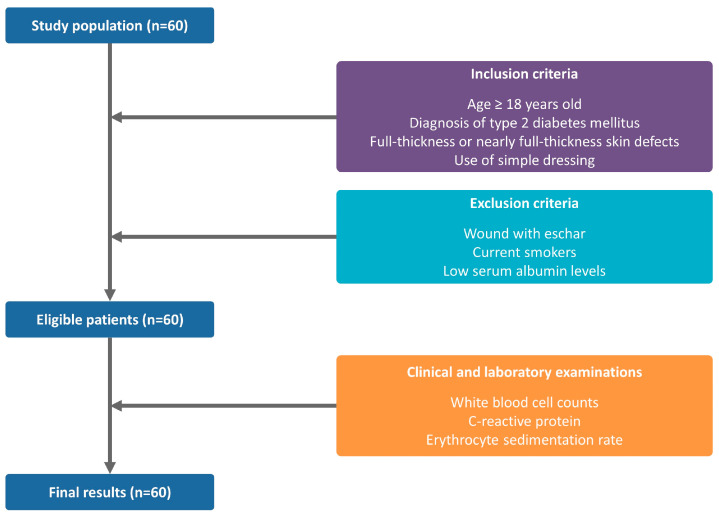
Study flow chart.

We examined a total of 60 patients (*n* = 60) with full- or nearly full-thickness skin defects. The cohort was composed of 30 men and 30 women, whose mean age was 48.3 ± 18.8 years old. The study flow chart is illustrated in Figure 2.

**Figure 3:**
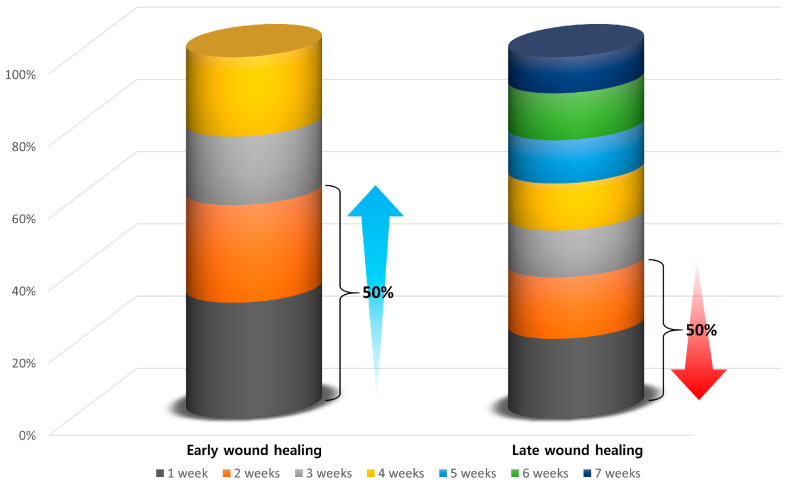
Early and late wound healing. Early wound healing (WH) was defined as a decrease in the total area of wounds by more than 50% within the first 2 weeks. In our series, there were 27 patients who achieved a greater than 50% decrease in the total wound area within the first 2 weeks and experienced wound closure within 5 weeks. Our clinical series of patients were therefore divided into the early WH group (*n* = 27) and the late WH group (*n* = 33).

In our series, there were 27 patients who achieved a greater than 50% decrease in the total area of wounds within the first 2 weeks and experienced wound closure within 5 weeks (Figure 3). Our clinical series of patients was divided into the early WH group (*n* = 27) and the late WH group (*n* = 33). Notably, BG and HbA1c levels were significantly lower in the early WH group as compared with its late counterpart (*p* < 0.001). Their baseline characteristics are represented in Table 1.

### 3.2. Wound Measurements

In our series, initial wound measurements included a surface area of 159.45 ± 179.26 mm^2^ and a length of wound edge of 11.67 ± 4.89 mm. The overall WHR was calculated as 3.73 ± 2.15 mm^2^/day.

**Figure 4:**
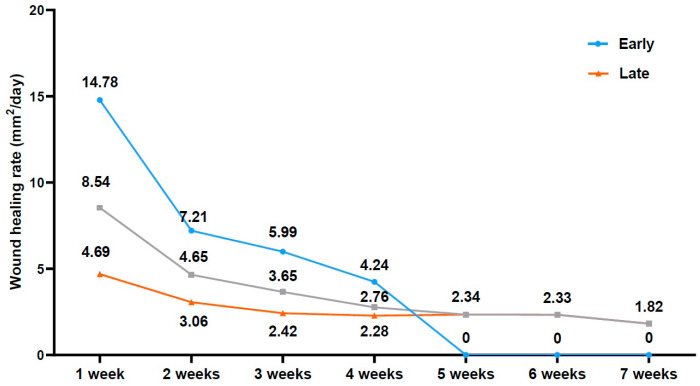
Time-dependent changes in the wound healing rate. After defining the wound healing rate (WHR) (mm^2^/day) as a daily decrease in the actual surface area of wounds, we monitored changes in the WHR during a 7 week period.

Time-dependent changes in the WHR during a 7-week period are shown in Figure 4. The WHR was 8.06 ± 4.02 mm^2^/day in the early WH group and 2.71 ± 0.88 mm^2^/day in the late group. This difference reached statistical significance (*p* < 0.001).

### 3.3. Correlations between Inflammatory Markers and WHR

Serum levels of inflammatory markers in each group are presented in Table 2. The table shows that WBC counts were significantly higher and serum levels of CRP and ESR were significantly lower in the early WH group than in the late group (*p* = 0.027, 0.036 and 0.043, respectively).

## 4. Discussion

According to a review of previously published studies of the exact molecular pathogenic and pathophysiologic mechanisms underlying impairment in WH processes, there are notable differences in biological features between normal and impaired WH. Pro-inflammatory responses to tissue injury are involved in the WH process, at a higher degree in chronic impaired wounds than in acute normal ones [35,36]. This is accompanied by a review article reporting that normal WH is characterized by the involvement of a single, transient tissue injury, limited stimulation of inflammatory cytokines, normal epithelialization, the synthesis of extracellular matrix (ECM), angiogenesis and the formation of scar tissue, while impaired WH is characterized by the involvement of a recurrent tissue injury leading to increased actions of pro-inflammatory cytokines [37].

In the current study, BG and HbA1c levels were significantly lower in the early WH group than in its late counterpart (*p* < 0.001). These results indicate that BG and HbA1c levels might serve as indicators of the WHR in diabetic patients. The relationship between BG or HbA1c levels and WHR has been well described in the literature. According to Christman et al., there was a daily decrease in WHR by 0.028 cm^2^ when there was an increase in HbA1c by 1.0%. Thus, these authors suggested that HbA1c, a measure of glycemia, might serve as a predictor of the WHR in diabetic patients [38].

Our results showed that serum CRP levels and ESR were significantly higher in the late WH group than in the early group, based on which it can be inferred that there is a significant inverse correlation between both inflammatory markers and WHR. This is in agreement with previously published studies in this series [26,39]. In the current study, we also found that WBC counts were significantly lower in the late WH group than in the early WH group. This is in agreement with a previous study showing that WBC helps to promote WH through the removal of debris [40].

There are diverse types of treatment modalities for patients with DWs, although they do not guarantee a rapid and definite repair process [41]. To date, diverse attempts have been made to develop alternative treatment options that are effective in promoting the WH process in patients with DM. Of these, only a few were found to be effective for the treatment of patients with DWs, although there is a growing demand for alternative treatment modalities using natural products [42]. Given this context, the antidiabetic effects of *Combretum molle* (*C. molle*) extract deserve special attention. Hamza et al. showed that *C. molle* inhibited pancreatic, dermal and deoxyribonucleotide (DNA) injury, and thereby improved the oxidative state in male rats, thus suggesting that the use of *C. molle* extract might be effective in ameliorating hyperglycemia [43]. Moreover, Hamza et al. also recommended the use of *C. molle* extract as a promising treatment option for patients with severe DFU [44].

Briefly, our results showed that WBC counts were significantly higher and serum levels of CRP and ESR were significantly lower in the early WH group as compared with the late group (*p* < 0.05). However, our results cannot be generalized, for several reasons:

(1) We evaluated only patients who were hospitalized at a single, secondary medical institution.
(2) We enrolled a small number of patients in the current study. We therefore cannot completely rule out the possibility of selection bias.
(3) We failed to consider the possibility that factors such as aging, obesity or exercise might affect the wound healing process. It is well established that the normal WH process may be impaired in elderly or obese individuals; these patients are susceptible to infection, prolonged pain and other complications [45,46]. Moreover, recent studies have suggested that physical exercise may promote the WH process even in the presence of aging and obesity [47].
(4) We failed to analyze the effects of confounding factors, such as hypertension, congestive heart failure or the location and shape of wounds, on WHR.
(5) We failed to perform a wound fluid assay of inflammatory cytokines and proteases involved in WH; proteases such as matrix metalloproteinase-2 and -9 (MMP-2 and -9) as well as inflammatory markers such as IL-1 and tumor necrosis factor-α (TNF-α) are significantly elevated in chronic wounds as compared to their acute counterparts, which has been well described in the literature [48].

Based on our results, it can be concluded that WBC as well as serum CRP and ESR levels showed significant correlation with the WHR in a cohort of patients with T2DM. However, further large-scale, multi-center studies using wound fluid assay are warranted to establish our results.

## Figures and Tables

**Table 1:** Baseline characteristics in each group.

Variables	Values		*p*-Value
	Early WH Group (*n* = 27)	Late WH Group (*n* = 33)	
Age (years)	45.2 ± 19.3	51.4 ± 18.3	0.208
Sex (male-to-female ratio)	14:13	16:17	0.128
Height (cm)	167.2 ± 8.3	161.6 ± 7.1	0.067
Weight (kg)	68.3 ± 10.2	62.9 ± 8.4	0.091
BMI (kg/m^2^)	27.3 ± 4.0	21.1 ± 2.2	0.113
BG (mg/dL)	189.3 ± 86.3	237.1 ± 72.5	<0.0001
HbA1c (%)	7.13 ± 2.5	9.22 ± 5.7	
**Underlying diseases**			
Hypertension	12 (44.4%)	19 (57.6%)	>0.05
Hepatitis	4 (14.8%)	4 (12.1%)	
Tuberculosis	2 (7.4%)	3 (9.09%)	
Stroke	4 (14.8%)	3 (9.09%)	
Chronic renal failure	3 (11.1%)	2 (6.06%)	
Congestive heart failure	1 (3.7%)	2 (6.06%)	
Verruca	1 (3.7%)	0 (0%)	
**Wound characteristics**			
Location			
Upper extremity	13 (48.1%)	16 (48.5%)	>0.05
Lower extremity	13 (48.1%)	15 (45.5%)	
Trunk	9 (33.3%)	11 (33.3%)	
Back	3 (11.1%)	3 (9.09%)	
Flank	1 (3.7%)	2 (6.06%)	
Chest	1 (3.7%)	1 (3.0%)	
Lower abdomen	1 (3.7%)	0 (0%)	
Shape			
Trapezoid	7 (25.9%)	9 (27.3%)	>0.05
Round	8 (29.6%)	8 (24.2%)	
Rod	5 (18.5%)	7 (21.2%)	
Irregular	5 (18.5%)	5 (15.2%)	
Triangle	2 (7.4%)	4 (12.1%)	
Surface area			
≥100 mm^2^	12 (44.4%)	15 (45.5%)	>0.05
<100 mm^2^	15 (55.6%)	18 (54.5%)	
**Bacterial infection**			
No growth	25 (92.6%)	30 (90.9%)	>0.05
MRSA	1 (3.7%)	2 (6.0%)	
*Staphylococcus epidermidis*	1 (3.7%)	1 (3.0%)	

WH, wound healing; BMI, body mass index; BG, blood glucose; MRSA, methicillin-resistant *Staphylococcus aureus*. Values are mean ± standard deviation or the number of cases showing the corresponding percentage, where appropriate. Statistical significance at *p* < 0.05.

**Table 2:** Serum levels of inflammatory markers in each group.

Variables	Values		*p*-Value
	Early WH Group (*n* = 27)	Late WH Group (*n* = 33)	
WBC (×10^6^/L)	15,300 ± 6400	8000 ± 5300	0.027 *
CRP (mg/L)	34.62 ± 13.75	146.28 ± 112.46	0.036 *
ESR (mm/h)	16.34 ± 5.26	29.14 ± 6.88	0.043 *

WH, wound healing; WBC, white blood cell count; CRP, C-reactive protein; ESR, erythrocyte sedimentation rate. Values are mean ± standard deviation. * Statistical significance at *p* < 0.05.

## Data Availability

The data presented in this study are available on request from the corresponding author. The data are not publicly available due to privacy reasons.
